# First-in-human validation of a DROP-IN β-probe for robotic radioguided surgery: defining optimal signal-to-background discrimination algorithm

**DOI:** 10.1007/s00259-024-06653-6

**Published:** 2024-02-20

**Authors:** Francesco Collamati, Silvio Morganti, Matthias N. van Oosterom, Lorenzo Campana, Francesco Ceci, Stefano Luzzago, Carlo Mancini-Terracciano, Riccardo Mirabelli, Gennaro Musi, Francesca Nicolanti, Ilaria Orsi, Fijs W. B. van Leeuwen, Riccardo Faccini

**Affiliations:** 1 National Institute of Nuclear Physics (INFN), Section of Rome, Rome, Italy; 2 Interventional Molecular Imaging Laboratory, Department of Radiology, Leiden University Medical Center, Leiden, The Netherlands; 3 Department of Scienze di Base e Applicate per l’Ingegneria (SBAI), Sapienza University of Rome, Rome, Italy; 4 Division of Nuclear Medicine, IEO, European Institute of Oncology IRCCS, Milan, Italy; 5 Department of Oncology and Hematology-Oncology, University of Milan, Milan, Italy; 6 Department of Urology, IEO European Institute of Oncology, IRCCS, Milan, Italy; 7 Department of Physics, Sapienza University of Rome, Rome, Italy

**Keywords:** Robotic surgery, Image-guided surgery, Molecular imaging, β radioguided surgery, Prostate cancer, PET

## Abstract

**Purpose:**

In radioguided surgery (RGS), radiopharmaceuticals are used to generate preoperative roadmaps (e.g., PET/CT) and to facilitate intraoperative tracing of tracer avid lesions. Within RGS, there is a push toward the use of receptor-targeted radiopharmaceuticals, a trend that also has to align with the surgical move toward minimal invasive robotic surgery. Building on our initial ex vivo evaluation, this study investigates the clinical translation of a DROP-IN β probe in robotic PSMA-guided prostate cancer surgery.

**Methods:**

A clinical-grade DROP-IN β probe was developed to support the detection of PET radioisotopes (e.g., ^68^ Ga). The prototype was evaluated in 7 primary prostate cancer patients, having at least 1 lymph node metastases visible on PSMA-PET. Patients were scheduled for radical prostatectomy combined with extended pelvic lymph node dissection. At the beginning of surgery, patients were injected with 1.1 MBq/kg of [^68^Ga]Ga-PSMA. The β probe was used to trace PSMA-expressing lymph nodes in vivo. To support intraoperative decision-making, a statistical software algorithm was defined and optimized on this dataset to help the surgeon discriminate between probe signals coming from tumors and healthy tissue.

**Results:**

The DROP-IN β probe helped provide the surgeon with autonomous and highly maneuverable tracer detection. A total of 66 samples (i.e., lymph node specimens) were analyzed in vivo, of which 31 (47%) were found to be malignant. After optimization of the signal cutoff algorithm, we found a probe detection rate of 78% of the PSMA-PET-positive samples, a sensitivity of 76%, and a specificity of 93%, as compared to pathologic evaluation.

**Conclusion:**

This study shows the first-in-human use of a DROP-IN β probe, supporting the integration of β radio guidance and robotic surgery. The achieved competitive sensitivity and specificity help open the world of robotic RGS to a whole new range of radiopharmaceuticals.

## Introduction

Being one of the most widely applied forms of image-guided surgery, radioguided surgery (RGS) is an interventional nuclear medicine technique that directs surgeons toward tissue targets preoperatively defined on imaging roadmaps such as PET/CT and SPECT/CT [1, 2]. To this aim, following initial diagnostics, a radiopharmaceutical is injected into the patient before surgery, and the surgeon is given an intraoperative detector enabling the real-time identification of areas with accumulated radiotracer.

Today, most RGS applications are based on the detection of low-energy (< 150 keV) γ-emitting radiopharmaceuticals (e.g., ^99m^Tc-based tracers). Localization of ^99m^Tc avid lesions is performed using intraoperative γ probes that provide numerical and acoustical feedback proportional to the counting rate and, thus, to the amount of radiopharmaceutical detected in the considered sample. A unique characteristic of the γ-based RGS approach is the long penetration of such radiation in human tissue. In fact, if we consider ^99m^Tc, 1/3 of its photons penetrate more than 8 cm of tissue. This property holds both advantages and disadvantages. It allows for the detection of “deeply located” lesions, but shine-through can pose a problem when the target tissue is located in an area characterized by elevated physiological uptake. Originally RGS was pursued in open surgery, but in recent years, detector technologies have adapted to support even robot-assisted surgery, e.g., via the DROP-IN γ probe [3, 4].

Since its introduction in the form of radioimmunoguided surgery (RIGS), tumor-targeted RGS is today undergoing a resurgence, a prime example being the introduction of PSMA-targeted surgery in prostate cancer patients [5]. Due to relatively low uptake values and more complex tracer pharmacokinetics, these receptor-targeted procedures have proven to be more challenging to perform than, e.g., sentinel lymph node (SLN) procedures [6]. Comparative analysis showed that signal-to-background ratios (SBRs) influence surgical decision-making in the context of RGS.

Given the focus of global radiochemistry efforts on the production of receptor-specific PET tracers, it makes sense to investigate if and how tracers already used in patients for PET diagnostics can also find use in RGS applications, with the aim of providing a complementary approach with respect to SLN procedures [6].

In this context, we have exploited the use of β particle detectors [7, 8]. Since β radiation undergoes more tissue attenuation, penetration is reduced to a few mms, and this feature could help mitigate the shine-through phenomenon [9]. The impact of this effect on SBR values builds on previous studies with the PET radiotracer [^68^Ga]Ga-PSMA using Monte Carlo simulations [10] and initial ex vivo evaluations [11]. In general, RGS is based on giving the surgeon real-time (numeric/acoustic) feedback of the amount of tracer that is accumulated in the area being sampled with the probe. However, especially for receptor-targeted procedures, only in very particular cases, this feedback is so unambiguous enough to allow immediate discrimination of tumors from healthy tissue. Indeed PET images as well as common clinical experience suggest that tissue uptake may vary significantly, not only between tumor and healthy tissue, but also among lesions. Discussion also remains on what should be defined as “background” during surgery [12]. Finally, there is also no agreement yet on what “a fixed, arbitrary” SBR cutoff should be defined to consider a tissue sample “positive” or “negative”; SBRs of 2 [13, 14] and 1.5 [15] were previously suggested for image guidance.

In the current study, we clinically translated a prototype DROP-IN β probe to investigate, for the first time, its in vivo use in prostate cancer patients. In particular, we studied how the technology can support the intraoperative decision-making process. Utilizing typical ROC curve analysis, we concentrated on the development of a statistically sound signal-to-background discrimination algorithm able to optimize the sensitivity and specificity of the tracing technique. This innovative approach was compared to the standard “fixed SBR method” that uses the cutoff values 1.5 and 2.

## Material and methods

### Engineering a clinical-grade DROP-IN β probe

**Fig. 1:**
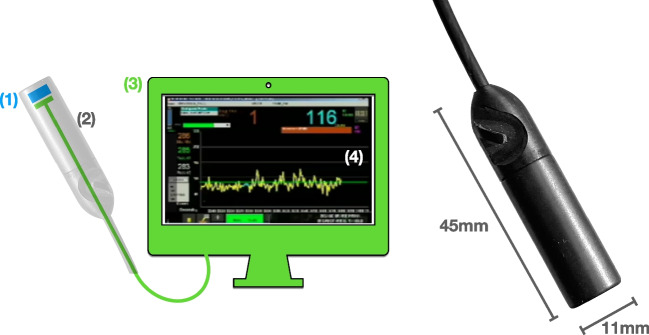
Left, scheme of the DROP-IN β probe components, as described in the text. Right, photo of the probe

The sterilizable DROP-IN set-up (see Fig. 1) consists of four main parts: (1) a β particle detector, (2) a DROP-IN housing, (3) an electronic processing and read-out unit, and (4) a dedicated statistical software algorithm for signal interpretation.

The core of the β detector itself consists of a cylindrical (6 mm diameter and 3 mm height) mono-crystalline para-terphenyl (doped with 0.1% in mass of (E,E)-1,4-diphenyl-1,3- butadiene) [16]. This material has been shown to be highly effective for β + detection, as it is transparent to the 511 keV γ particles. Tests with ^68^ Ga source suggest that it has an ~ 80% efficiency to positrons above ~ 110 keV while being, at the same time, substantially transparent to photons of ~ 500 keV, with an efficiency of ~ 3% [7, 17]. This intrinsic γ transparency therefore limits the need for collimation. The scintillation crystal is surrounded by a 1-mm-thick external black polyvinyl chloride (PVC) ring, combined with the housing described below.

Scintillation light conversion was performed by a 3 × 3 mm^2^ silicon photomultiplier (SiPM Hamamatsu S13360-3050PE) powered and read-out by a custom microcontroller, connected to the device with a biocompatible and sterilizable latex-free cable.

The DROP-IN housing hosting the crystal and the SiPM was designed to be picked up with the robotic steerable instruments (e.g., ProGrasp Forceps, Intuitive). The DROP-IN shape and diameter (11 mm) were sized to make it compatible with typical trocar dimensions (maximum 12–15 mm). Length (45 mm) and weight (6 g) were optimized to also facilitate maneuverability.

The housing was realized in PEEK (polyetheretherketone): a light, water-tight, sterilizable, robust, and biocompatible plastic material. To meet the required light tightness, black PEEK was chosen. The housing lateral wall thickness was 1.5 mm, while β-detection sensitivity at the front of the detector was maximized by machining the PEEK wall to be as thin as 300 µm.

The electronics then translate the analog signal produced by β particle crossing into both numerical (i.e., counts/s) and audible feedback. Exposure time (or counting time) was chosen as 1 s.

Since the definition of the most optimal statistical algorithm to guide surgical decision-making was, as already described, one of the aims of this study, no further visual information was shown to the surgeon during the operation, apart from a strip chart of the acquired countings (see Fig. 1).

### Patients’ population and surgical procedure

The clinical study was performed at IEO Milano (Institutional Review Board of the European Institute of Oncology, protocol code UID 1703, clincialtrial.gov ID NCT05596851) and considered 7 primary prostate cancer patients, scheduled for radical prostatectomy combined with extended pelvic lymph node dissection. All patients had high-risk prostate cancer according to EAU classification, a tumor stage of cT3 and Gleason scores between + 3 and 4 + 5, and a positive diagnostic PSMA-PET/CT scan with at least 1 lymph node metastasis visible. To enable β RGS, all patients received a second low-dose injection of [^68^Ga]Ga-PSMA-11 (about 1.1 MBq/kg) directly in the operating room prior to surgery.

The surgical resection was performed with a da Vinci Xi robotic surgical platform, and lymph node dissection preceded the prostatectomy. Beta probe scanning/tracing was performed, following the template, on a district basis. A district is defined as an area sharing the same definition of tracer background. For each district scanned, the surgeon was instructed to follow this procedure:

1. Perform a background acquisition in the given district. The probe was used to measure background for at least 5–10 s, keeping it in contact with the tissue, in an area assumed free of disease.
2. Use the probe to analyze different lymph nodes in the considered district according to the followed surgical template, with the possibility to perform more than one measurement over the same sample.

The surgeon was left free to decide at their judgment how many times and where to perform background acquisitions, for example, when moving to another, distant zone or if a significant time had elapsed.

To easily compare measurements acquired at different times, all probe counts were corrected for the physical decay of ^68^ Ga. Only lymph nodes were analyzed with the probe, and all in vivo measurements were recorded for offline investigation (raw data + da Vinci video + probe video). To ensure no positive lesions were missed, every excised sample was checked ex vivo with a second β probe based on the same technology used for the DROP-IN prior to sending it out for standard pathological examination.

### Signal-to-background ratio discrimination algorithm

In a previous ex vivo study on GEPNET samples, using [^90^Y]Y-DOTATOC, a statistical approach for the discrimination algorithm was proposed and tested, leading to a sensitivity of 93% and specificity of 100% [18, 19]. Here, the cutoff was defined as follows:

1 $${R}_{{\text{Cutoff}}}=<{{R}_{{\text{Bkg}}}}^{{\text{Flat}}}>+{N}_{\sigma }\times {\sigma }_{{\text{Flat}}},$$

where $<{{R}_{Bkg}}^{{\text{Flat}}}>=({{R}^{{\text{Bkg}}}}_{{\text{Max}}}+{{R}^{{\text{Bkg}}}}_{{\text{Min}}})/2$ is the expected value of background countings, assuming them to have a flat distribution, ${\sigma }_{{\text{Flat}} }=({{R}_{{\text{Bkg}}}}^{{\text{Max}}}-{{R}_{{\text{Bkg}}}}^{{\text{Min}}})/\sqrt{12}$ is the corresponding variance, and ${N}_{\sigma }$ is chosen to be 3.

In the higher background context faced by β^+^-RGS, using [^68^Ga]Ga-PSMA, such a discrimination algorithm becomes more complex, as the challenge is to identify efficiently tumor samples while avoiding accidental background signals of 511 keV γ rays.

In this study, we set out to identify and optimize the best way to adapt this cutoff approach, considering two possible ways a positive lesion can present.

A sample is rated “Probe-Positive” if either one of these two conditions is satisfied:

2 $${\text{Frac}}=\frac{{N}_{{\text{AboveCutoff}}}}{{N}_{{\text{Tot}}}}\ge {\epsilon }_{{\text{Frac}}},$$

where ${N}_{{\text{AboveCutoff}}}$ is the number of countings above a threshold ${R}_{{\text{Cutoff}}}$, ${N}_{{\text{Tot}}}$ is the total number of countings in the sample, and ${\epsilon }_{{\text{Frac}}}$ is a parameter of the algorithm to be defined or

3 $${N}_{{\text{AboveCutoff}}}^{{\text{Contiguous}}}\ge {\epsilon }_{{\text{Num}}},$$

where ${N}_{{\text{AboveCutoff}}}^{{\text{Contiguous}}}$ is the length of the longest series of contiguous countings exceeding ${R}_{{\text{Cutoff}}}$ and ${\epsilon }_{{\text{Num}}}$ is another parameter of the algorithm.

In order to fully exploit the discriminating power of the probe, ${R}_{{\text{Cutoff}}}$ is defined as in Eq. 1 but leaving ${N}_{\sigma }$ free to vary.

**Fig. 2:**
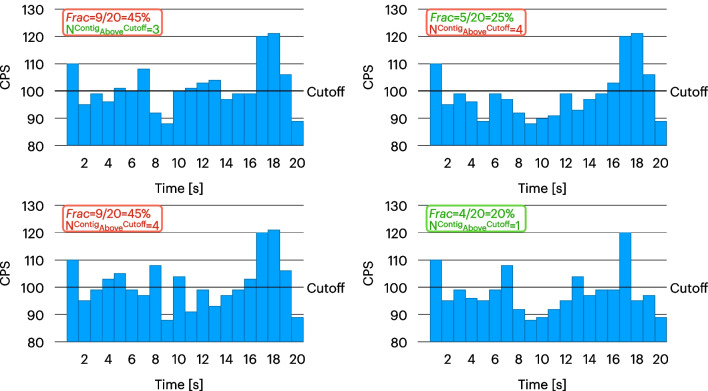
Effect of the two detection conditions (Eqs. 2 and 3). In this example, a 20 s acquisition was performed on a sample, having a cutoff value of 100 CPS (horizontal line), considering the algorithm parameters: ${\upepsilon }_{{\text{Num}}}=4$ and ${\upepsilon }_{{\text{Frac}}}=30\mathrm{\%}$. The color of the legend text suggests whether the two detection conditions were satisfied (red, indicating tumor) or not (green, indicating free of tumor). A red outline of the total legend means that the algorithm identified the sample as Probe + (indicating tumor)

How these two conditions allow to identify possible lesions having different countings topologies is clarified in Fig. 2.

To find the best possible settings of this approach, thus delivering the best sensitivity and specificity as compared to pathological results, the combination of these 3 parameters was optimized:

- The number of sigmas above the mean value we set the cutoff to, ${N}_{\sigma }$ (see Eq. 1) that we varied to assume the values {0.5, 1, 1.5, 2, 2.25, 2.5, 2.75, 3, 3.25, 3.5, 3.75, 4, 4.5, 5, 6, 8, 10, 30} (${{N}^{{\text{Values}}}}_{\sigma }=18$)
- The minimum fraction of countings above a signal in a sample ${\epsilon }_{{\text{Frac}}}$ (see Eq. 2) that we varied in the ensemble {5%, 10%, 20%, 50%} (${{N}^{{\text{Values}}}}_{{\epsilon }_{{\text{Frac}}} }=4)$
- The minimum number of consecutive countings above a threshold ${\epsilon }_{{\text{Num}}}$ (see Eq. 3) that we allowed to assume the values {2, 3, 4, 6, 8, 10, 20} (${{N}^{{\text{Values}}}}_{{\epsilon }_{{\text{Num}}} }=7)$

All things considered, for each sample, the same data were analyzed with a combination of 504 (${{N}^{{\text{Values}}}}_{\sigma }\times {{N}^{{\text{Values}}}}_{{\epsilon }_{{\text{Frac}}} }\times {{N}^{{\text{Values}}}}_{{\epsilon }_{{\text{Num}}}}$) different parameters, for each of which the sample could be defined as “Probe-Positive” or “Probe-Negative.” This result was then compared with pathological analysis, considered as the standard reference, defining each sample as Path.-Positive or Path.-Negative and thus allowing to classify it as true positive (TP), true negative (TN), false positive (FP), or false negative (FN).

Therefore, for each chosen value *N*_σ_, sensitivity and specificity can be calculated as.

$${\text{Sensitivity}}=\frac{{N}_{{\text{um}}}{T}_{{\text{rue}}}{P}_{{\text{ositives}}}}{{{N}_{{\text{um}}}T}_{{\text{rue}}}{P}_{{\text{ositives}}}+{{N}_{{\text{um}}}F}_{{\text{alse}}}{N}_{{\text{egatives}}}},$$

$${\text{Specificity}}=\frac{{{N}_{{\text{um}}}T}_{{\text{rue}}}{N}_{{\text{egatives}}}}{{{N}_{{\text{um}}}T}_{{\text{rue}}}{N}_{{\text{egatives}}}+{{N}_{{\text{um}}}F}_{{\text{alse}}}{P}_{{\text{ositives}}}},$$

And, for a given pair of ϵ_Frac_ and ϵ_Num_, a ROC curve can be constructed by plotting the variation of sensitivity and specificity with *N*_σ_.

To strengthen the analysis, the whole dataset was split into two parts: one to be used for training the algorithm (training dataset) and the other to test it (test dataset). To this aim, data were randomly split on a district basis and assigned to either the training or test dataset, in order to find a distribution of districts between the two samples that contain, according to pathology, a similar number of healthy and diseased areas.

## Results

### DROP-IN probe: clinical translation and data collection

**Fig. 3:**
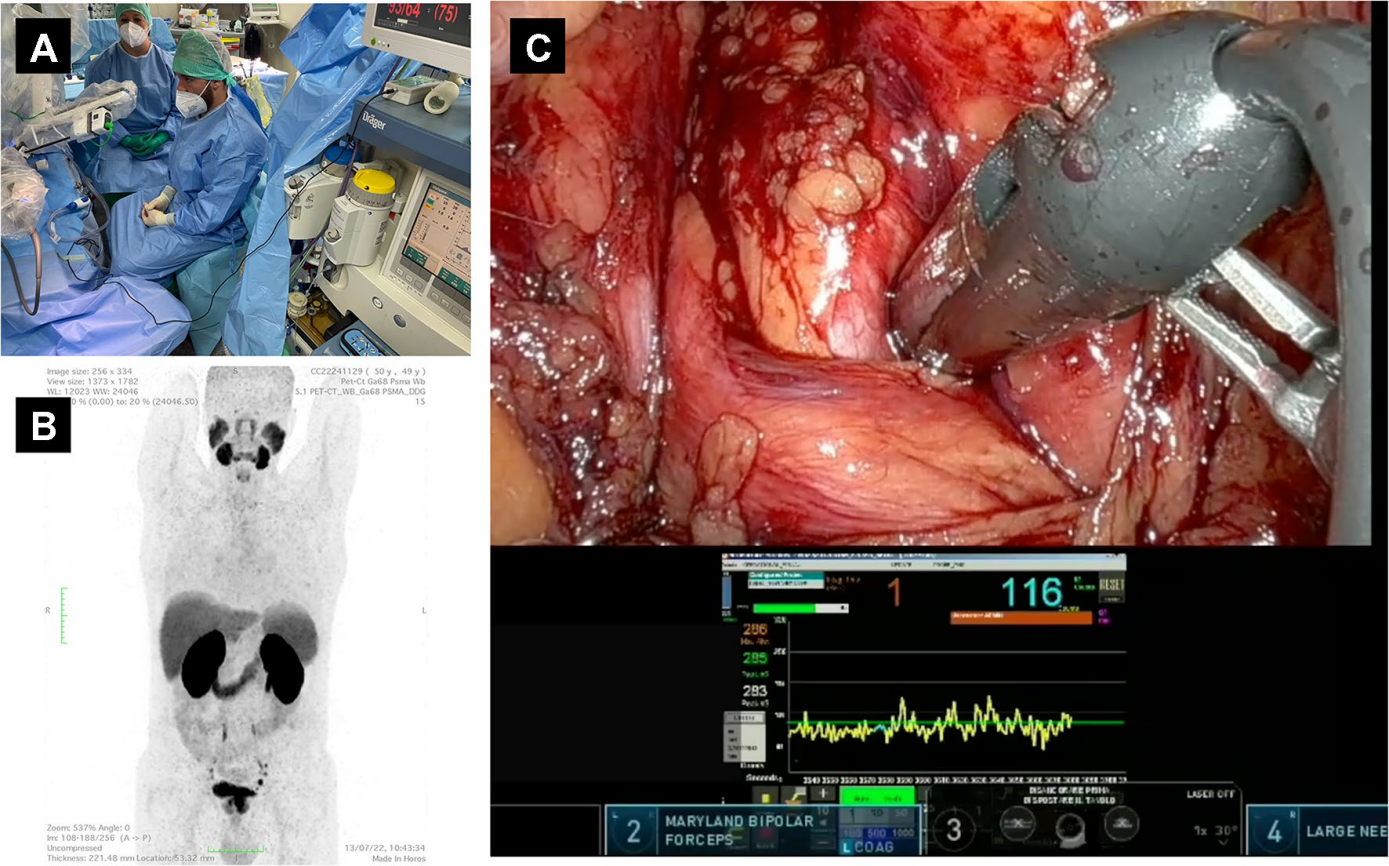
**A** Operating room during one of the procedures: the black cable coming from the drop-in probe can be distinguished. **B** MIP image of ^68^ Ga-PSMA-PET of one of the considered patients (Pt. #3). **C** View of the da Vinci monitor as seen by the surgeon during the procedure (Pt. #5), including the TilePro split-screen option to view the probe measurements directly in the surgical console

Before every procedure, the medical grade DROP-IN β probe was sterilized with a standard gas-plasma sterilization cycle. During the surgical procedures, it was used through a standard 12-mm assistant trocar (see Fig. 3). Pick-up of the DROP-IN β probe was simple using the standard da Vinci tools (e.g., ProGrasp Forceps instrument). The surgeon was able to use the probe to scan the field autonomously exploiting all 6 available degrees of freedom, without the need of any help from the assistant. In each patient, the DROP-IN probe was used to perform lymph node examination for around 20 min in total, having therefore reduced impact on surgery duration. No complications related to the use of the prototype probe nor of the [^68^Ga]Ga-PSMA tracer were observed, and no issues were encountered in the sterilization procedure.

Seven patients were considered in this study, and surgery was performed between Jan 2023 and Oct 2023. Median injected activity was 100 MBq (IQR 84–107). Probe measurements were performed in a time after injection ranging from 20 to 152 min (median 49 min, IQR 34–94). A total of 66 samples were analyzed with the probe in vivo, belonging to 25 districts (i.e., a group of samples for which the surgeon retained to use the same background evaluation). Of these 66 samples, pathology examination identified 31 to be bearing tumors (47% “Path.-Positive”) and 35 not (“Path.-Negative”), while only 19 were PET-positive and 47 PET-negative. Patients’ data are summarized in Table 1.

**Table 1:** Patient data

Variables	Values
Preoperative data	
Patients included	7
Age (median (IQR), y)	63 (53–68)
PSA (median (IQR), g/mL)	10 (8.4–20)
BMI (median (IQR) kg/m^3^)	23.5 (23–28.5)
Tumor staging	CT3N1M0 for all pts
PSMA-PET-positive lymph nodes*, *n* (median per pt., IQR)	19 (3 (1.5, 3))
Intraoperative data	
Analyzed and resected samples, *n* (median per pt., IQR)	66 (10 (7.5, 10.5))
Probe-positive samples, *n* (median per pt., IQR)**	28 (4 (2,5))
Pathology data	
Tumor-positive lymph nodes, *n* (median per pt., IQR)	31 (5 (3, 5.5)
Probe versus pathology	
Sensitivity (%)**,***	76%
Specificity (%)**,***	93%

*According to ePSMA criteria

**Based on the best signal-to-background cutoff algorithm investigated below

***On the test dataset

### Signal-to-background cutoff value: identification and testing

**Fig. 4:**
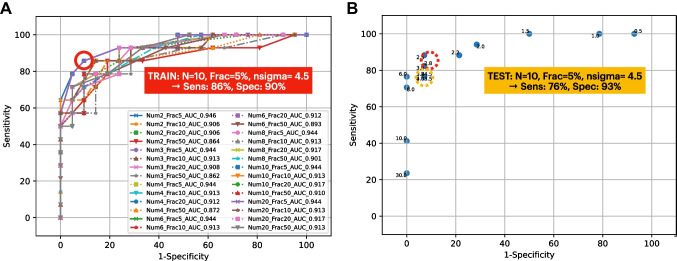
**A** ROC curves obtained as described in the “Signal-to-background ratio discrimination algorithm” section for the train dataset. The circled point represents the most effective cutoff, corresponding to ${\upepsilon }_{{\text{Num}}}=10$, ${\upepsilon }_{{\text{Frac}}}=5\mathrm{\%}$, and ${N}_{\upsigma }=4.5$. **B** ROC curve obtained for the test dataset, in the case identified as the most effective cutoff in the train dataset (whose sensitivity and specificity are also shown for reference in the red circle). The yellow-circled point represents the performances of this same cutoff in the test dataset. Numbers nearby points refer to the nsigma of the given point

As described in the “Signal-to-background ratio discrimination algorithm” section, the surgically collected dataset was used to statistically calculate the best SBR discrimination algorithm for surgery, i.e., the one having the most optimal sensitivity and specificity as compared to pathology. To this end, the dataset was randomly split in such a way as to have two sub-datasets comprising a similar amount of positive and negative samples. In particular, among all possible combinations, the train dataset contained a total of 35 samples, while the test dataset contained 31 samples. The procedure described in the “Signal-to-background ratio discrimination algorithm” section was then applied to the train dataset: 28 (= ${{N}^{{\text{Values}}}}_{{\upepsilon }_{{\text{Frac}}} }\times {{N}^{{\text{Values}}}}_{{\epsilon }_{{\text{Num}}}}$). ROC curves, having 18 (= ${{N}^{{\text{Values}}}}_{\sigma }$) points each, were constructed (see Fig. 4A).

All ROCs were found to have a high area under the curve (AUC) (median 0.911, IQR 0.905–0.921). The best point in this curve, representing the most effective cutoff on this train dataset, is circled in Fig. 4A and was chosen as the one nearest to the 100 − 100 reference value. This was found to be the point relative to ${\epsilon }_{{\text{Num}}}=10$, ${\epsilon }_{{\text{Frac}}}=5\%$, and ${N}_{\sigma }=4.5$, having sensitivity = 86% and specificity = 90%.

Figure 4B compares this best-performing point in the train dataset (red circle) with the corresponding value in the test dataset (yellow circle), found to have 76–93% sensitivity–specificity. Considering the relatively small amount of data points available for this analysis after the splitting, similar values for sensitivity and specificity were therefore found between the two datasets, thus confirming the robustness of the identified cutoff and more generally of the defined approach.

### Application of the best cutoff value algorithm

**Fig. 5:**
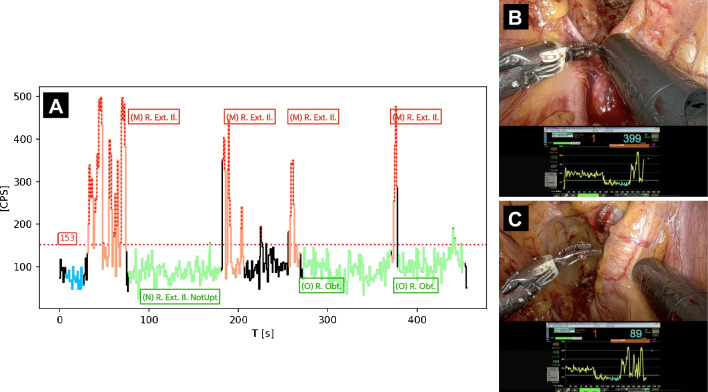
**A** Example of data acquired when scanning a given district with the best discrimination algorithm. Blue data represent counts considered to evaluate the background, while orange and green ones represent measurements performed on tumor and healthy tissue respectively, according to pathology examination. The label reports the name and pathology code of the considered sample, while the color of its surrounding border represents whether the sample was identified as probe-positive (red) or probe-negative (green) by the algorithm. **B**, **C** Screenshots of the DROP-IN probe performing measurements on samples M and N respectively

To demonstrate how the suggested signal-to-background cutoff algorithm could assist surgical decision-making in future in vivo studies, Fig. 5 visualizes a typical acquisition of a district in the current surgical dataset, using the best algorithm parameters. In the plot, blue data represent the background measurement phase, while orange and green data represent probe measurements performed respectively on Path.-Positive and Path.-Negative samples. In this specific district example of patient #2, the cutoff value (red horizontal dashed line) was found to be *R*_Cutoff_ = 153 CPS. All countings exceeding this cutoff are red dashed, but only samples also complying with “condition formula 2” or “condition formula 3”, with ${\epsilon }_{{\text{Num}}}=10$, ${\epsilon }_{{\text{Frac}}}=5\%$, are considered probe-positive and have therefore a red border around their label. In the particular case represented in Fig. 5, therefore, thanks to the discrimination algorithm, the probe would have identified 1 true positive and 2 true negatives.

Applying the best cutoff algorithm to the entire patient data obtained in this study provides the following clinical outcome: the probe was able to detect 15 out of 19 PSMA-PET-positive lesions (78% detection rate). However, from the 4 missed, two turned out to be PET false positives at pathology, increasing the probe detection rate to 88% compared to PET, while the remaining two were most probably missed due to the presence of healthy tissue between them and the probe in the surgical setting. Interestingly, the probe did find 10 true positive samples, based on pathology, that were not found on preoperative PET. Even if a detailed correlation of probe counting with sample dimension was outside the scope of this first study, and therefore, not all samples’ footprints were measured; the ones being PET − but Probe + were found to have a diameter of 3–4 mm. When taking pathology as the true standard, the probe sensitivity and specificity were found to be 81% and 91% respectively considering the whole dataset, values that are as expected in line with those found in train and test datasets.

**Fig. 6:**
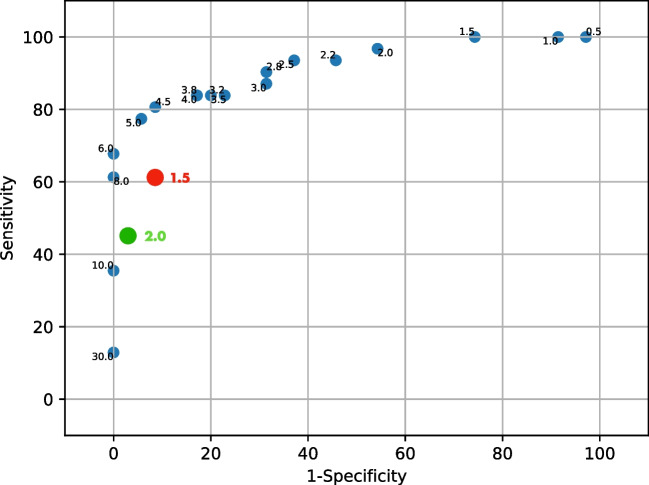
Comparison of sensitivity and specificity achievable, in the whole data sample, via the discussed statistical algorithm with respect to the one obtained by following the “fixed SBR” approach. The two most commonly used SBR values (1.5 and 2) have been considered for this comparison

To compare the performances of the investigated discrimination algorithm with the typical “fixed SBR cutoff approach,” sensitivity and specificity were also calculated for a fixed 1.5 SBR cutoff ratio (60 and 90%, respectively) and 2 SBR cutoff ratio (45 and 97%, respectively; see Fig. 6).

## Discussion

We investigated the first-in-human translation of a medical grade DROP-IN β probe, a technology that supports the detection of β + emitting tracers, so-called “PET tracers” [20], such as [^68^Ga]Ga-PSMA-11, thereby extending the impact of the respective PSMA-PET imaging roadmaps on surgical interventions. A recent EANM position paper indicates that technical advancement is needed to fully exploit beta-RGS [21], and a Delphi study on PSMA-targeted surgery indicates that more education on this topic is desirable [22]. These are critical points that we address in our report.

In PSMA cancer management PSMA-targeted RGS provides a complementary value to SLN-RGS procedures, allowing targeting of both macro- and micro-metastases [6], concepts that rely heavily on the DROP-IN design. Similar to what was previously reported for the DROP-IN γ probes [23], the DROP-IN β probe helped to fully exploit the degrees of freedom of steerable robotic instruments. Since background and target signal intensities affect intraoperative decision-making and surgical performance [24], we used our first-in-human data to define and optimize a statistical software algorithm providing a quantified signal cutoff value that could support surgical decision-making. While a fixed SBR value is often utilized for this application [12], this approach lacks a real statistical foundation and quantified and sound reasoning as to which SBR value should be chosen in radioguided surgery applications. After optimization, the proposed statistical algorithm for the identification of probe-positive lymph nodes outperformed the use of SBR cutoffs of either 1.5 or 2, when considering both sensitivity and specificity. A limitation of the current study is the limited number of samples on which the algorithm was trained and applied, in particular, due to the need to split the dataset for training and testing purposes. The reproducibility of our results between the two independent sub-samples suggests that the high sensitivity and specificity values are not incidental. Ongoing studies focus on the optimization of the proposed cutoff algorithm to the clinical settings using a larger patient cohort and thus a larger amount of positive and non-positive lymph nodes. It could be investigated if the algorithm is transferable to γ-RGS as well or if it should be redefined for every procedure (i.e., a combination of probe, tracer, and surgical application). Lastly, further optimization of the technique will allow to tailor the amount of injected activity to the given case, thus reducing it further with respect to the current “half PET” starting value.

It needs to be highlighted that demonstrating clinical benefit was not in the scope of this initial first-in-human study. However, a recent systematic review on PSMA-targeted surgery [12] indicates that different groups are pursuing different strategies to surgically identify PSMA-positive lesions. To assess the impact of the technology presented in this study, it is critical to compare the sensitivity and specificity to other studies that investigated PSMA-targeted surgery in the primary setting [13, 25–28]. Table 2 indicates that despite the ~ 3-mm-range tissue penetration of the DROP-IN β radiation, its sensitivity for lymph node detection seems to be at least as good as optimized near-infrared fluorescence guided surgery approaches which are assumed to support a larger signal penetration of < 10 mm. This is a rather unique finding, especially when one considers that the β-guidance approach requires about 300 times less of a PSMA-targeting tracer and that this can be the exact same tracer already used in the clinic for PSMA-PET. Combined, these features would help reduce costs and stimulate the dissemination of the image guidance concepts to hospitals that have access to [^68^Ga]Ga-PSMA-PET. The performance with β-guidance even equaled some of the γ-radioguidance examples, a finding that indicates that limiting the shine-through of background signals with beta detectors could provide a competitive edge.

**Table 2:** Robotic PSMA-targeted surgery in primary prostate cancer—limited metastatic lymph node detection

Reference	Detection modality	# of patients	# of Lymph node samples	Dosing	Time to surgery	Sensitivity	Specificity	False positives	False negatives
Stibbe et al. [28]	Fluorescence (λ ex = 775 nm, λ em = 795 nm)	4*	20	30 μg/kg	24 h	0%	100%	0	2
Nguyen et al. [27]	Fluorescence (λ ex = 774 nm, λ em = 793 nm)	6*	80	25 μg/kg	24 h	71%	97%	6	2
Gandaglia et al. [13]	γ (99 mTc; 140 keV)	12	256	~ 0.15 μg/kg**	20 h	63%	99%	1	3
Gondoputro et al. [26]	γ (99 mTc; 140 keV)	12	74	~ 0.1 μg/kg**	18 h	76%	96%	2	5
Yılmaz et al. [25]	γ (99 mTc; 140 keV)	15	297	~ 0.15 μg/kg**	17 h	100%	100%	0	0
This study	β + (68 Ga; 1.9 MeV)	7	66	~ 0.1 μg/kg***	0.5 h	76%	93%	6	3

*Different dosings included in these studies, but this was the optimized dosing

**Estimated from [14]

***Estimated from [29]

The current study opens up a whole world of possible minimal invasive RGS applications [20]. Firstly, [^68^Ga]Ga-PSMA-guided surgery can be further explored, for example, also in the salvage setting for prostate cancer. There are then nevertheless also other possible options, like FDG-guided surgery (e.g., lung cancer [30]), carbonic anhydrase iX guided surgery (e.g., renal cell carcinoma [31]), C-MET guided surgery (e.g., renal cell carcinoma [32]), folate guided surgery (e.g., lung cancer [33]), and fibroblast-activation-protein guided surgery (i.e., > 28 different cancer types [34]).

To widen the technological impact, the use of 18F-based RGS is being investigated in parallel. Hereby, the results from a study that uses [^18^F]F-FDG in recurrent cervical cancer are encouraging [35]. Applications are expected to improve following technical refinements such as the exploitation of solid-state detectors that are more effective in combination with low-energy positrons as emitted by 18F, with respect to [^68^Ga] [36–38].

## Conclusion

We show for the first time the clinical translation of a DROP-IN β probe that supports RGS in a robotic surgery setting. The obtained sensitivity and specificity values for nodal metastases were found to be competitive to values obtained for other PSMA-targeted surgery strategies.

## Data Availability

The datasets generated during and/or analyzed during the current study are available from the corresponding author on reasonable request.
